# “Focused Ultrasound-mediated Drug Delivery in Humans – a Path Towards Translation in Neurodegenerative Diseases”

**DOI:** 10.1007/s11095-022-03185-2

**Published:** 2022-03-07

**Authors:** Joanna M. Wasielewska, Anthony R. White

**Affiliations:** 1 Cell & Molecular Biology Department, Mental Health Program, QIMR Berghofer Medical Research Institute, Brisbane, QLD Australia; 2 Faculty of Medicine, The University of Queensland, Brisbane, QLD Australia; 3 School of Biomedical Sciences Faculty of Medicine, The University of Queensland, Brisbane, QLD Australia

**Keywords:** Blood-brain barrier, Neurodegenerative disease, Focused ultrasound, Drug delivery, Human-induced pluripotent stem cell

## Abstract

The blood-brain barrier (BBB) has a major protective function in preventing the entry of harmful molecules into the brain, but is simultaneously limiting the delivery of drugs, restricting their potential clinical application in neurodegenerative diseases. Recent preclinical evidence demonstrates that following application of focused ultrasound with microbubbles (FUS+MB), the BBB becomes reversibly accessible to compounds that normally are brain-impermeable, suggesting FUS+MB as a promising new platform for delivery of therapeutic agents into the central nervous system. As a step towards translation, small cohort clinical studies were performed demonstrating safe BBB opening in Alzheimer’s disease, Parkinson’s disease and amyotrophic lateral sclerosis (ALS) patients following FUS+MB, however improved drug delivery has not yet been achieved in human. Simultaneously, rapid progress in the human induced pluripotent stem cell (hiPSC) modeling technology allowed for development of novel Alzheimer’s disease patient-derived BBB *in vitro* model that reacts to FUS+MB with BBB opening and can be used to answer fundamental questions of human BBB responses to FUS+MB in health and disease. This review summarizes key features of the BBB that contribute to limited drug delivery, recapitulates recent advances in the FUS+MB mediated human BBB opening *in vivo* and *in vitro* in the context of neurodegenerative disorders, and highlights potential strategies for fast-track translation of the FUS+MB to improve bioavailability of drugs to the human brain. With safe and effective application, this innovative FUS+MB technology may open new avenues for therapeutic interventions in neurodegenerative diseases leading to improved clinical outcomes for patients.

## Introduction

### The Blood-brain Barrier as a Major Obstacle to Drug Delivery in Neurodegenerative Diseases

The blood-brain barrier (BBB) formed in all cerebral capillaries is a dynamic multicellular interface that controls the exchange of molecules between the blood and the brain parenchyma [1]. By separating the circulating blood from the brain, it protects the central nervous system (CNS) from harmful molecules and pathogens, while at the same time maintaining tightly regulated brain homeostasis. Although essential for brain functioning, the BBB limits the ability of therapeutic agents to penetrate into the CNS, representing a major challenge in the treatment of neurodegenerative disorders [2].

The BBB is primarily formed by the specialised brain microvascular endothelial cells (BMECs) closely connected through tight junctions (TJs), adherent junctions (AJs), and gap junctions (GJs) [1]. At the molecular level, those junctional complexes are organised into sophisticated structures involving transmembrane proteins (such as claudins, occludins and junctional adhesion molecules (JAMs)) and numerous auxiliary proteins (as zonula occludens proteins, ZO-1, ZO-2 and ZO-3) that link adjacent BMECs and limit the para-cellular permeability of the BBB [1]. In contrast to other vascular endothelial cells found in the peripheral organs, specialised BMECs lack fenestrations (with only ~ 4 nm wide extracellular gaps limited by TJs being present between BMECs as compared to 50 nm wide intracellular gaps in peripheral endothelium). The BBB endothelial cells also significantly restrict transcytosis (with BMECs containing scarce 1–15 vesicles/μm^2^ compared to 30–40 vesicles/μm^2^ in the peripheral EC), further contributing to minimal permeability of the BBB [3–6].

Other specialised components of the BBB include pericytes, astrocytes, adjacent neurons and non-cellular basement membrane, which together with BMECs form a so-called neurovascular unit (NVU) [1]. All elements of the NVU play an essential role in maintaining the BBB integrity and function. Pericytes are a member of the vascular smooth muscle cell lineage that regulate flow of blood in brain capillaries and contribute to the maintenance of TJs and AJs between adjacent BMECs. Astrocyte end-feet cover up to 99% of the BMEC surface and secrete glial-derived factors (such as glial cell line-derived neurotrophic factor (GDNF)) and morphogens (as retinoic acid or sonic hedgehog (Shh)) which support BMECs and improve the tightness of the BBB [7, 8]. The BBB is also in close connection with dendrites originating from local neurons that participate in the neurovascular coupling – a process essential for adequate blood supply to brain regions of increased neural activity. Finally, cells forming the NVU are embedded in the basement membrane – a unique form of thick extracellular matrix formed by fibronectin, collagen type IV, laminin and proteoglycan that supports the integrity and signalling at the BBB. These described properties and multicellular organisation result in a very high trans-endothelial resistance of the BBB reaching up to 5,900 Ohm/cm^2^ *in vivo* (as compared to 20 Ohm/cm^2^ measured in peripheral capillaries), forming one of the tightest barriers in the body [9–11].

Therefore, physiologically, only O_2_, CO_2_, water and small lipophilic molecules can pass through the BBB *via* simple diffusion, whereas the transport of most molecules is regulated by the expression of selective transporters at the surface of the BMECs [2]. Examples include solute carrier (SLC) transporters such as GLUT-1 (a glucose transporter), LAT-1 (an amino acid transporter), DMT-1 (divalent metal transporter 1) or SMVT (sodium dependent multivitamin transporter) that allow for controlled transport of essential nutrients, vitamins and ions between the blood and the CNS [12]. Importantly, BMECs are also equipped with specialised efflux transporters that actively remove harmful metabolites and xenobiotics from the BMEC cytoplasm back to the blood, but simultaneously prevent the majority of drugs from entering the CNS. Correspondingly, expression of over 15 drug efflux transporters have been identified in BMECs, including multidrug resistance transporter (MDR1) and P-glycoprotein protein (P-gp), which together with the TJs, limit effective transport of therapeutics targeting the brain [13–16]. Finally, BMECs contain a variety of drug-metabolizing enzymes, that degrade or chemically modify drugs leading to their inactivation [17]. The aforementioned structural and molecular characteristics of the BBB significantly restrict successful drug delivery in the context of brain disease, limiting therapeutic potential of promising preclinical drug candidates. Importantly, vast evidence indicates that different mechanisms of molecular transport at the BBB may be altered during neurodegeneration, which further affects the distribution of therapeutics in the brain and decreases treatment efficiency [18]. Therefore, the development of innovative drug delivery methods is an urgent medical need to facilitate successful outcomes in treating neurodegenerative disorders.

## Focused Ultrasound as an Innovative Approach to Opening the Blood-brain Barrier

**Fig. 1:**
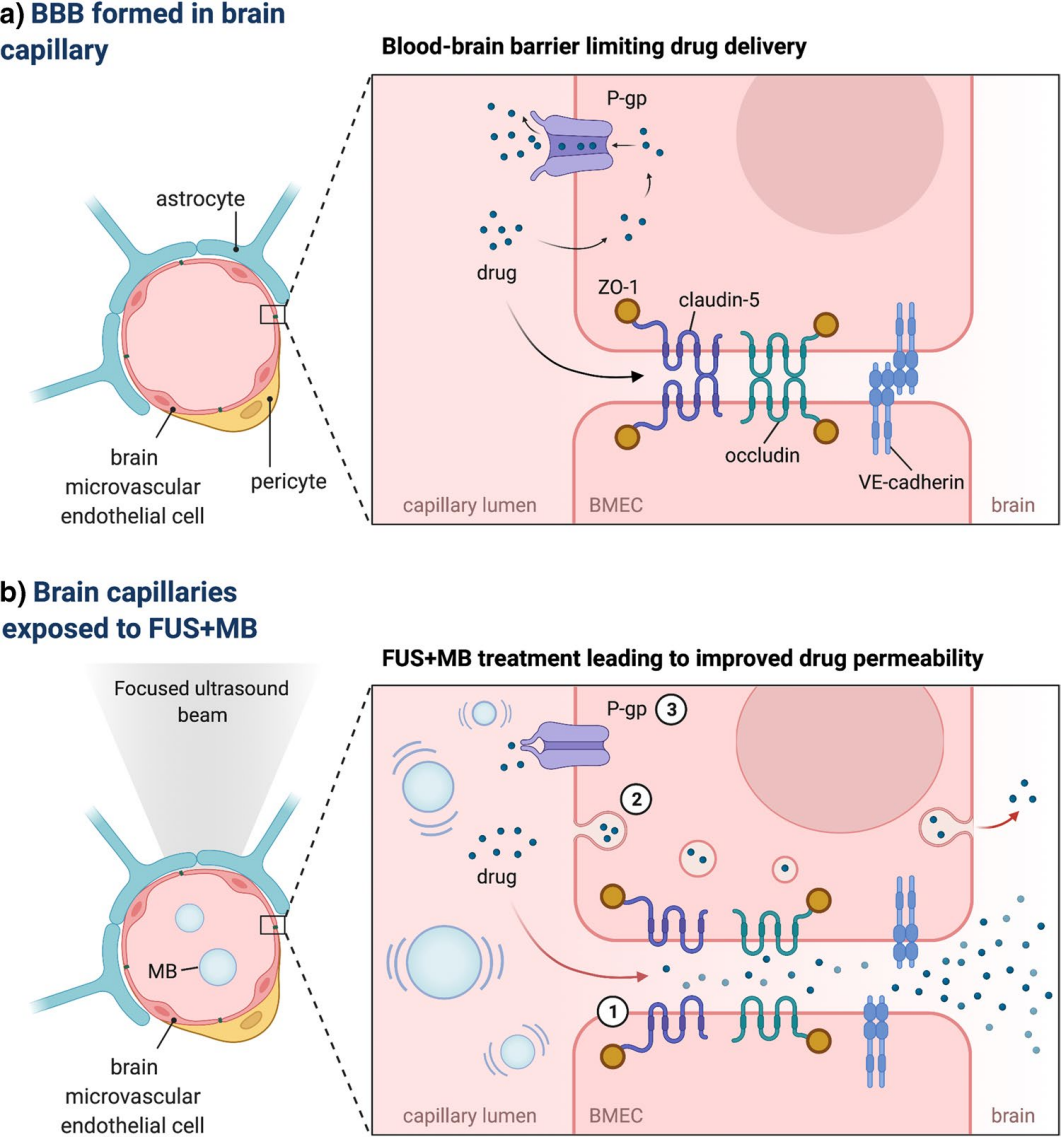
**Proposed mechanisms of focused ultrasound and microbubble mediated drug delivery at the blood-brain barrier. (a)** Physiologically, the blood-brain barrier (BBB) formed by brain microvascular endothelial cells (BMEC), pericytes and astrocytes restricts the permeability of delivered drugs from the blood into the brain. The presence of tight- and adherens junctions containing transmembrane adhesion proteins such as vascular endothelial (VE)-cadherin, occludin, claudin-5 and zonula occludens-1 (ZO-1) accessory protein prevents paracellular transport of most hydrophilic molecules. Efflux transporters as P-glycoprotein (P-gp) actively remove a wide range of drugs from the BMEC cytoplasm, including small (< 400 Da) lipophilic molecules that might otherwise passively diffuse across BMEC to brain parenchyma. Transcytosis of molecules through the BBB is largely limited, significantly impeding the entry of therapeutic agents into the brain. (**b)** Preclinical observations identify three proposed routes of focused ultrasound and microbubble (FUS+MB) mediated drug delivery at the BBB. (**1**) MB oscillating in the ultrasonic field produce mechanical forces on the of tight- and adherens junctions, leading to temporal junction opening and improved paracellular transport of delivered drug. (**2**) FUS+MB treatment causes increase in the number of intracellular vesicles and upregulation of endo- and transcytosis at BMEC suggesting stimulation of transcellular transport at the BBB. (**3**) Exposure to FUS+MB temporarily (48-72 h) suppresses expression of P-gp at BMEC, potentially limiting drug efflux at the BBB. [1, 28, 31, 32, 118, 119]. BBB-blood-brain barrier; BMEC- brain microvascular endothelial cell; FUS+MB- focused ultrasound and microbubble; P-gp- P-glycoprotein; ZO-1-zonnula occludens; VE-cadherin- vascular endothelial cadherin; Figure created with BioRender.com.

With the BBB being the major challenge in the delivery of therapeutics for treating CNS disorders, several different strategies have been proposed to overcome this structural and functional barrier. One of them is an application of focused ultrasound (FUS) which, in combination with gas-filled microbubbles (MB), leads to reversible BBB opening and consequent improvements to drug bioavailability in the brain (Fig. 1). Since the initial discovery of its biological effect [19], vast progress has been made in the preclinical validation and technical development of FUS, leading to the initiation of clinical trials investigating application of therapeutic ultrasound in neurodegenerative disorders. Given the important advantages of FUS over other drug delivery methods such as non-invasiveness, spatial and temporal precision, reversibility and promising additional therapeutic effects [20–23], FUS holds the potential to emerge as an innovative multimodal tool for the treatment of neurodegenerative disorders.

### Current Understanding of Focused Ultrasound and Microbubble Effects on Brain Vasculature

**Fig. 2:**
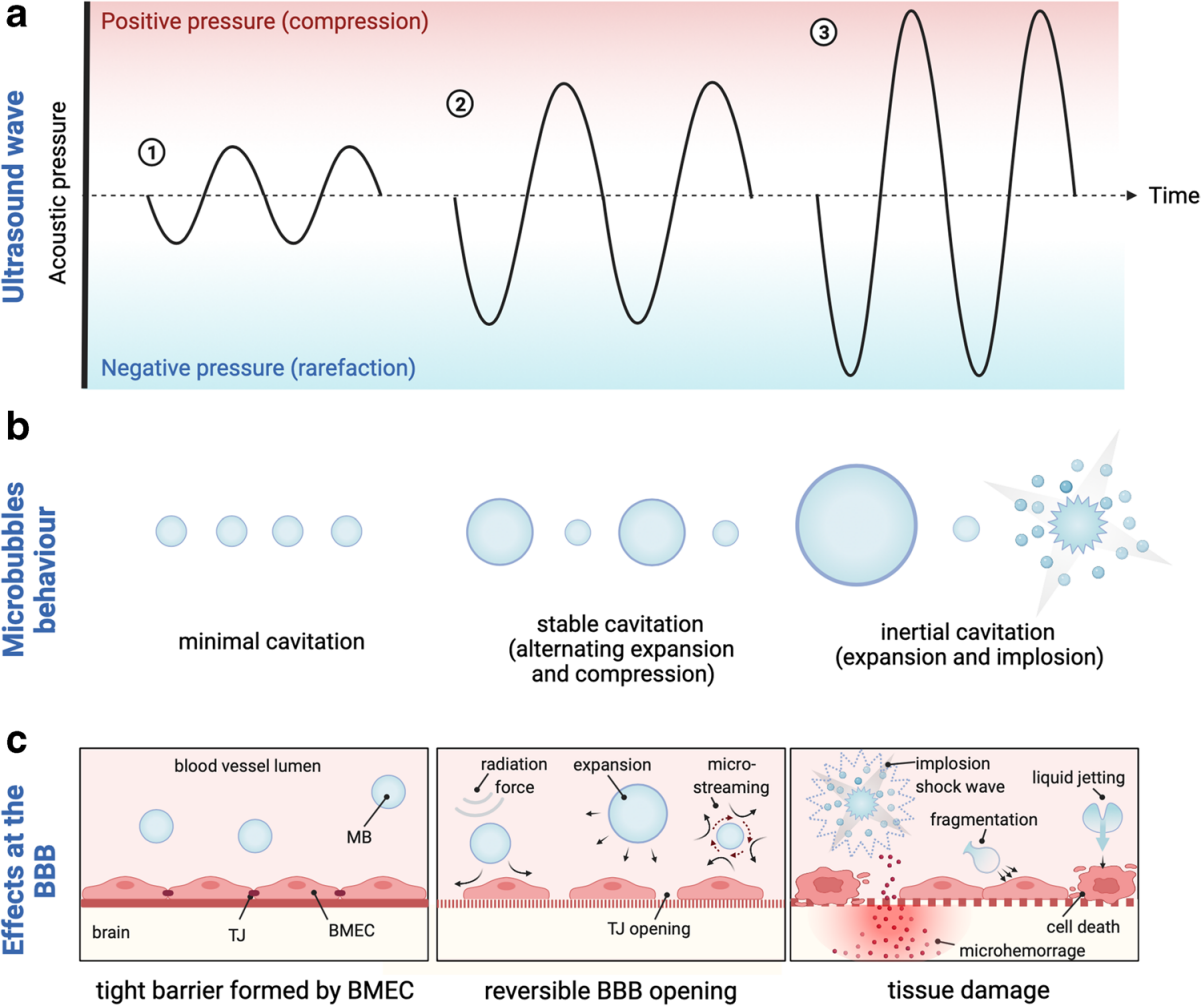
**The interplay between focused ultrasound, microbubbles and cerebral vasculature.** Physical interactions between ultrasonic wave (**a**) and microbubbles (MB) (**b**) determine the bioeffects at the blood-brain barrier (BBB) (**c**). When exposed to the ultrasound wave, MB decrease in diameter during the compression portion of the wave and increase during the rarefaction phase. (**1**) Ultrasound applied at insufficient acoustic pressure causes minimal volumetric oscillations of MB and the BBB remains closed. (**2**) Optimal ultrasound acoustic pressure induces stable MB contraction and expansion (stable cavitation) that exerts mechanical forces on brain microvascular endothelial cells (BMEC), leading to reversible BBB opening. Linearly cavitating MB generate the flow of liquid/blood around themselves (microstreaming) that in turn produces sheer stress on BMEC membrane, causing increased BBB permeability. Expanding MB create tension at tight junction (TJ) proteins leading to junction opening. Acoustic radiation force propels oscillating MB to the BMEC layer, further enhancing MB and BBB interactions. (**3**) At higher acoustic pressures, MB collapse violently, producing shock waves and micro-jets. This abrupt inertial cavitation generates strong mechanical stress at the BBB leading to permanent TJ disruption, irreversible BMEC membrane perforation, microhemorrhage and tissue necrosis. [24, 25, 27, 33–35]. BBB-blood-brain barrier; BMEC- brain microvascular endothelial cell; MB-microbubble; TJ-tight junction; Figure created with BioRender.com.

Successful clinical application of FUS+MB requires an in depth understanding of how its physical parameters translate to the desired biological effect in the human brain. Current evidence coming from preclinical studies suggests that the BBB opening effect is achieved by the complex interactions of microbubbles and the vasculature in the ultrasonic field, with the extent of opening dependent on applied ultrasound parameters (frequency, acoustic pressure, burst repetition frequency, burst length, sonication duration) and microbubble properties (chemical formulation, concentration, size distribution, half-life) [24–27] (Fig. 2). When exposed to the ultrasound at low pressure, MB are known to oscillate volumetrically (expand and contract) in response to cycles of compression and rarefaction. This MB behavior termed stable cavitation produces mechanical effects on BMECs lining brain capillaries, including microstreaming (which is streaming flow of fluid/blood around an oscillating MB) and increased shear stress, that in turn creates tension at TJs and leads to increased vascular permeability [24]. Oscillating MB have also been shown to activate BMEC cell surface receptors and downregulate the expression of efflux transporters (such as P-gp), and therefore modulate transcellular transport of large molecules at the BBB [28–32]. Ultrasound applied at excessive acoustic pressure however, causes rapid collapse of MB (termed as inertial cavitation) that can generate strong mechanical stresses, MB micro-jetting and thermal effects in the vasculature. This uncontrolled MB disruption underlies the majority of adverse effects observed during FUS-mediated BBB opening, including micro-hemorrhages, edema, glial scarring or tissue necrosis [33–35].

Given that MB behavior, and resulting bioeffects, are strongly dependent on the properties of the ultrasound wave, an in-depth understanding of sonication parameters is likely to drive safer application of FUS in humans. As such, although ultrasound frequencies ranging from 28 kHz to 8 MHz have been used to successfully to open BBB in animal models, for application in human brain the upper limit of 1.5 MHz has been proposed to offer optimal focal volume of BBB opening with minimal (or no) focus aberration and tissue damage [36–38]. Secondly, given that the MB expansion and inertial cavitation are directly dependent on the peak positive and negative acoustic pressure (PNP) of the insonating wave, multiple studies investigated the relationship between PNP and vascular permeability [39, 40]. It has been shown that below a certain threshold, MB radial expansion and vascular leakage are positively correlated with PNP, however, further increase of the PNP leads to excessive inertial cavitation of the MBs and corresponding tissue damage [40, 41]. Although the interspecies differences (e.g. in skull acoustic attenuation) complicate direct translation of preclinical FUS parameters to human brain, it is now accepted that increasing acoustic pressure modulates the degree of BBB opening and/or adverse effects on the vasculature [42], and FUS at pressures at 0.2 - 1.0 MPa (220 kHz) have been safely applied in small cohort clinical studies [43–46]. Finally, FUS burst-mode scheme including burst length, burst repetition frequency and sonication duration, although receiving less attention, have all been shown to affect biological responses to FUS+MB [47]. In this regard, a study presented by McDannold *et al*. reported positive correlation between FUS burst length and BBB permeability for bursts lasting 0.1 – 10 ms [24]. A further increase of burst length beyond 10 ms did not lead to increased BBB opening, possibly due to complete collapse of all MBs before the end of each burst [24, 48]. Interestingly, increasing burst repetition frequency from 0.1 Hz to 1 Hz, and therefore decreasing 10-fold the sonication time needed to deliver the same number of bursts, also led to permeability enhancement [49, 50]. This effect has been attributed to the short half-life of MBs in the circulation (estimated ~ 1.3 min for commercially available MB) and correspondingly reduced MB decay and larger number of MBs simultaneously oscillating during shorter FUS treatments. Importantly, Choi *et al*. observed also that the extent of BBB opening is affected by the length of the resting periods, where FUS bursts schemes including resting periods shorter than 0.1s or longer than 1s (for 1.5 MHz frequency) produced no effects on BBB permeability [24, 49].

Considerable progress has also been made in understanding how inherent MB properties affect the extent of BBB opening. Several studies reported a relationship between MB diameter and BBB permeability enhancement, where larger MBs were shown to induce a higher degree of BBB opening resulting in longer recovery time post treatment [51–53]. The extent of BBB opening was also positively correlated with the concentration of applied MBs [54]. Interestingly, Tung *et al.* showed that size-sorted MB with a diameter of 6-8 μm required lower acoustic pressure (0.3 MPa, 1.5 MHz), compared to MB with a diameter of 1-2 μm, which required a higher acoustic pressure (0.45 MPa, 1.5 MHz) to induce the same degree of BBB disruption [55]. Similar effects were observed by Choi *et al*. demonstrating close interplay between MB properties and applied FUS wave characteristics [56]. From a clinical perspective, all described parameters are of great relevance for successful and controlled drug delivery in the context of potential treatment for neurodegenerative disease.

## Focused Ultrasound Application in Neurodegenerative Disease Patients

Despite extensive understanding of disease pathology and discovery of promising preclinical drug-candidates, brain disorders associated with profound neurodegeneration, including Alzheimer’s disease (AD), Parkinson’s disease dementia (PD) and amyotrophic lateral sclerosis (ALS), still have no cure. A primary obstacle linking all these disorders is the presence of the BBB limiting the transport of up 98-100% of administered drugs, preventing them from reaching therapeutic concentrations in the brain [57]. To overcome this hurdle, FUS+MB technology was recently evaluated in the first small cohort clinical studies opening a new avenue of investigation in the treatment of neurodegenerative diseases.

Lipsman *et al.* was the first to show BBB opening in a cohort of five AD patients using focused ultrasound and MB treatment [44]. In this seminal study, magnetic resonance-guided FUS (MRgFUS) was applied twice over the period of one month, targeting dorsolateral prefrontal cortex. Although no beneficial effects were found in amyloid burden or cognitive scores following sonication, the authors successfully demonstrated reversible and repeatable BBB opening in the absence of any adverse effects. Interestingly, transient changes in the resting state functional connectivity were observed post MRgFUS in patients, confirming possible neuromodulatory effects of FUS+MB reported in animal studies [21, 23, 58]. This pioneering work was followed by Rezai *et al.* who achieved FUS-mediated opening of the BBB in hippocampus and entorhinal cortex (EC) of six AD patients [59]. Enrolled patients tolerated a total of 17 MRgFUS treatments with no adverse neurological or cognitive effects, demonstrating FUS+MS as a mean of non-invasive, reproducible, transient and spatially precise BBB opening. Interestingly, a follow up study revealed amyloid-β plaque reduction following FUS+MB, suggesting promising therapeutic effects of FUS+MB in the absence of any additional drug treatment [60]. Subsequently, a clinical trial led by Mehta *et al.* confirmed feasibility of BBB opening in AD patients and identified a perivenular immunologic healing response downstream from the FUS+MB application, expanding our understanding of how FUS affects human vasculature [61].

Importantly, application of MRgFUS is expanding to other neurodegenerative diseases, with Abrahao and colleagues recently demonstrating the first evidence of successful BBB disruption in the motor cortex of four ALS patients [43] and Gasca-Salas *et al*. reporting safe BBB opening in PD, targeting the right parieto-occipito-temporal cortex [45]. In both studies, no adverse side-effects were observed, with mild cognitive improvement being recorded post-treatment in PD patients. Interestingly, authors of all described clinical trials reported spontaneous closure of the BBB within 24 h post opening, suggesting 24 h as a clinically relevant drug administration time-window, applicable to various types of neurodegenerative disorders.

### Technical State-of-the-art of Blood-brain Barrier Opening in Human

**Fig. 3:**
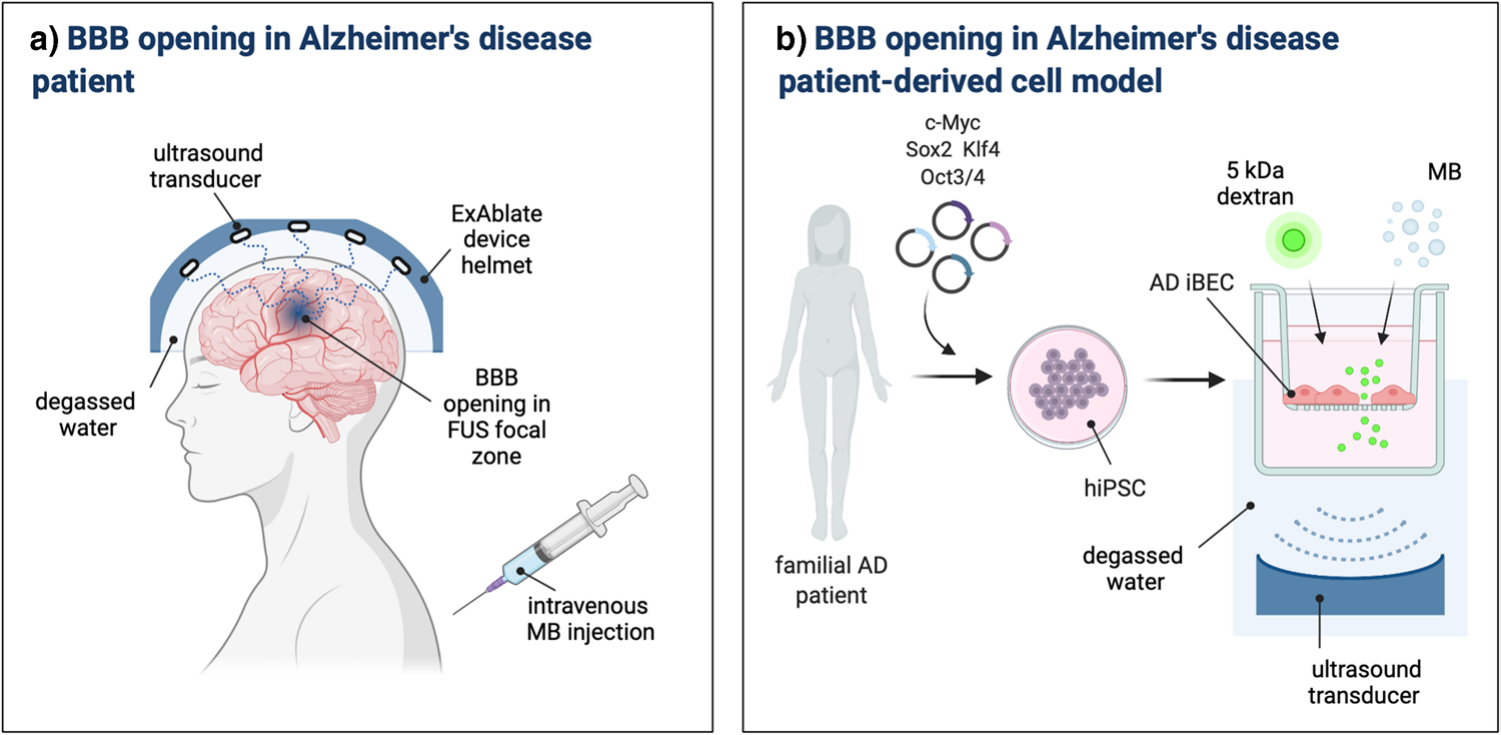
**Schematic representation of blood-brain barrier opening in Alzheimer’s disease patient** ***in vivo*** **and patient-derived model** ***in vitro*.** (**a**) Schematic of a magnetic resonance (MR)-guided ExAblate device used in the first successful blood-brain barrier (BBB) opening in Alzheimer’s disease (AD) patients. System consists of a hemispherical helmet lined with >1000 independent transducer elements delivering low frequency ultrasound treatment to the prescribed target. The helmet is positioned in the specialised MRI bed with stereotaxic frame and the space between patient’s head and the helmet filled with degassed water for acoustic coupling. Microbubble (MB) administration is carried out using repeated bolus injection or a continuous infusion. Reversible BBB opening occurs in the defined ultrasound focal zone. (**b**) Schematic of AD patient-derived human BBB opening *in vitro*. Somatic cells (e.g. fibroblasts or blood cells) are obtained from familial AD patients and reprogrammed to human induced pluripotent stem cells (hiPSC) by introduction of cocktail of reprogramming factors. hiPSC are used to generate brain endothelial-like cells (iBEC) and develop patient-derived *in vitro* AD BBB model. *In vitro* BBB is exposed to focused ultrasound and MB in the degassed water, leading to BBB opening and improved permeability of 5 kDa dextran. [44, 84]. AD-Alzheimer’s disease; BBB-blood-brain barrier; FUS-focused ultrasound; hiPSC-human induced pluripotent stem cell; iBEC- brain endothelial-like cells; kDa-kilodalton; MB-microbubble; Figure created with BioRender.com.

Promising clinical trial outcomes have motivated simultaneous technological progress leading to the development of clinical FUS brain devices and MB formulations. Currently available clinical prototype devices include extracorporeal low-frequency (220-230 kHz) MR-guided ExAblate Neuro Type 2 (InSightec, Israel) and neuronavigation-guided NaviFUS (Taiwan), as well as skull-implantable SonoCloud (CarThera, France), all allowing for non-thermal MB-mediated treatments [44, 62–64]. Although overcoming the problem of an ultrasound wave attenuation by the human skull, SonoCloud is currently the most invasive treatment. In this approach, the planar FUS transducer needs to be placed into a bur hole made on the patient’s skull, targeting only one specific brain region that may not reflect more diffuse neurodegenerative disease pathological targets. Thus, the SonoCloud approach may be primarily limited to brain cancer treatment involving highly localized application [62, 65]. The ExAblate Neuro device, which has proven successful in all clinical trials to date [43–45, 59], integrates 1024 individual transducers with a frequency of 220 kHz into a hemispherical helmet (Fig. 3a). Depending on the selected brain target location, the patient’s skull properties, intraoperative MRI data, helmet orientation, and additional information (such as no-pass zones predicted for each patient), the system determines the necessary parameters for each individual transducer element to deliver precise FUS treatment to a prescribed target location. The device also integrates a dedicated single-element acoustic detector, allowing for real-time MB cavitation monitoring to support selection of sonication parameters for each individual procedure. The disadvantage of the system is that it requires intraoperative magnetic resonance imaging (MRI) to provide treatment guidance, therefore increasing associated cost, time and invasiveness for the patient.

Given physical and cognitive symptoms associated with neurodegenerative diseases and late disease onset for most of dementias, outpatient, non-invasive and relatively short FUS procedures must be considered to deliver treatment to the vast majority of patients. To achieve this goal, a frameless NaviFUS device (NaviFUS Inc.) incorporating a neuronavigation system is being currently tested, and has showed initial promising outcomes in recurrent glioblastoma patients [64]. Neuronavigation allows the guidance of the invisible FUS focal beam without intra-procedural imaging, as the patient’s previous MRI or CT data are used to interactively visualize the position of applied FUS on a 3D anatomical brain image [64, 66]. This promising technology has been approved for first clinical trials targeting Alzheimer’s disease, possibly providing evidence of its utility in neurogenerative disorders. Additional FUS-devices originating from academic settings are also undergoing preclinical validation, expanding future treatment possibilities [67–69].

Microbubble formulations available in the clinic include FDA approved Definity (Lantheus Medical Imaging, USA), Optison (GEHealthcare,USA), and SonoVue (Bracco, Italy). The commercial MBs are sized 1-3 μm, 2-4 μm, and 2-5 μm respectively, offer a therapeutic application window of 5-10 min, and have different compositions and concentrations, allowing for control over desired biological effects. For example, larger MBs (i.e. SonoVue) were found to have a longer effective circulation time, facilitating extended sonication that may be required to deliver larger drugs such as therapeutic antibodies, however they were also associated with a higher risk of hemorrhage in preclinical studies [52, 54, 55, 70, 71]. Currently, the majority of clinical studies investigating effects of FUS in neurodegenerative diseases reported successful usage of lipid-shelled Definity (ExAblate) and SonoVue (NaviFUS) [44, 64], however continuous preclinical efforts in the MB development may lead to safer and more effective therapeutic applications of MB for BBB opening in patients.

With the safety window being sensitive to a multitude of technical factors, interdisciplinary research integrating biological and engineering perspectives may pave the way towards wide clinical application of FUS in neurodegenerative diseases.

## Focused Ultrasound Application in Human Blood-brain Barrier *in vitro* Models

Despite the promising clinical success of BBB opening in AD, ALS and PD, FUS+MB mediated drug delivery has not yet been trialed in patients due to many unanswered questions concerning interactions of human vasculature, ultrasound, and related drug transport dynamics.

An important step towards understanding the response of the human BBB to FUS+MB and accurate modeling of improved drug transport in various diseases may come from *in vitro* BBB models derived from human induced pluripotent stem cells (hiPSC). BBB models derived from hiPSCs have recently been shown to closely reflect *in vivo* drug permeability in the human brain [72, 73]. With increasing availability of hiPSC lines generated from fibroblasts of patients suffering from neurodegenerative disorders and carrying disease-associated mutations (e.g. in genes including *PSEN1* and *APOE E4* in AD [74, 75], *C9orf72* and *SOD1* in ALS [76], *LRRK2* and *SNCA* in PD [77]) and constantly improving hiPSC differentiation protocols [78–83], *in vitro* BBB models may become a useful tool to study FUS-mediated drug transport. In fact, a recent study presented by Oikari *et al.* achieved human BBB opening *in vitro* with focused ultrasound, applying clinically relevant FUS parameters (Table 1) [84]. The authors developed an *in vitro* AD BBB model based on hiPSC-derived induced brain endothelial cells (iBEC) that responded to FUS+MB treatment with reversible BBB opening and increased permeability to a small molecule fluorescent tracer (Fig. 3b). Furthermore, iBECs carrying an AD-associated *PSEN1* mutation showed longer recovery post treatment, identifying the first clinically relevant genotype-related difference in responses of human BBB to FUS+MB. Although drug delivery has not yet been trialed in this *in vitro* platform, promising results obtained with a 5 kDa cargo molecule suggest suitability of this system for future semi-high throughput drug permeability screening.

**Table 1:** Clinically Relevant Ultrasound and MB Parameters for Blood-brain Barrier Opening in Neurodegenerative Disease Patients and *in vitro* Model of Neurodegeneration. [43–45, 84]

Parameter	*In vivo* human patients	*In vitro* human BBB model
Center frequency	220 – 230 kHz	286 kHz
Peak negative pressure	0.2 – 1.0 MPa	0.15 – 0.3 MPa
Mechanical index	0.4	0.3 – 0.6
Burst length	10 ms	20 ms
Sonication duration	50 s – 120 s	120 s
Microbubble dose	4-10 μL/kg (Definity)	10 μL/well (in-house MB)
Reference	[43–45]	[84]

BBB- blood-brain barrier; kHz- kilohertz; MB- microbubble; MPa-megapascal; ms- millisecond; s- second;

Finally, extensive research indicates that pathological changes at the BBB in neurodegenerative diseases could have implications for drug transport [1]. Therefore, prior to broad clinical application, it is important to understand how FUS+MB could modulate the leakage kinetics of drugs in cerebral vasculature of patients suffering from distinct disorders. Patient-derived cells forming the NVU have been shown to maintain disease-associated phenotypes (BMECs [76, 84], pericytes [85], astrocytes [74, 86–88]). Therefore, investigation of FUS effects using *in vitro* models could help to optimise parameters accommodating pathological changes at the BBB characteristic to specific disease and/or genotype, and understanding molecular mechanisms responsible for FUS-mediated drug transport in brain dis orders. Application of FUS+MB to *in vitro* BBB models could also facilitate the analysis of sonication-associated effects at single cell resolution, otherwise not possible in the living human brain. Furthermore, with hundreds of potential new-drug candidates being investigated in pre-clinical and early clinical trials for the treatment of various neurodegenerative disorders, effective high-throughput *in vitro* screening methods are necessary to identify therapeutics most compatible with FUS-mediated delivery in humans. This in-depth understanding of patient-specific cellular and molecular responses to FUS may aid in the selection of the drug candidates and FUS parameters most suitable for specific patients and disease.

## Human *in vitro* BBB Models can Bridge the Gap Between FUS-mediated Drug Delivery in Preclinical Animal Models, and Successful Clinical Therapeutic Outcomes

It is estimated that the brain capillary network in humans has a total length of 600 km, providing 15-30 m^2^ surface of selectively permeable BBB and offering a highly attractive route of drug delivery into the brain in the context of disease [89].

FUS+MB has extensively shown to increase permeability of therapeutic agents in animal models of neurodegeneration including AD [90–93] and PD [94–97], leading to the reversal of some disease symptoms. With initial clinical trials showing safe BBB opening in humans [43–45], currently the biggest challenge is to use FUS+MB to achieve improved drug delivery in human patients in the absence of any adverse effects.

One of the first steps to facilitate this is to induce opening of the larger focal volume of the BBB in neurodegenerative disease patients. Currently, successful clinical trials report opening of >1 cm^3^ of human BBB [43, 44], which given widespread pathology observed in neurodegenerative diseases, may not be sufficient to achieve meaningful therapeutic outcomes. However, since FUS+MB has been shown to induce proinflammatory responses in animal models [98] and age-associated blood-borne factors could be detrimental if entering the brain in excessive amounts [99, 100], opening of the BBB in the entire brain as achieved in FUS-scanning mode in mouse models [20, 92, 101] can have adverse effects for the patient.

hiPSC-derived BBB models may become an important tool for simulation of potential adverse responses in human BBB to FUS+MB, and identify co-treatments or settings that could attenuate these effects. The high-throughput potential of novel human BBB models [83, 102] and applicability of FUS+MB *in vitro* [84] could also facilitate screening of various combinations of disease-targeting drug and adjuvant therapies alleviating effects of FUS in the context of distinct neurodegenerative diseases or underlying mutations, before clinical application. For example, glucocorticoids (GC) have known immunosuppressive effect and were shown to reduce brain edema and improve tightness of the BBB by upregulating expression of occludin, claudins and VE-cadherin [103]. Since iBECs carrying AD-associated *PSEN1* mutation previously demonstrated prolonged recovery post FUS *in vitro* [84], GC could be trialed to reduce inflammation and support barrier closure in familial AD patients who could be at risk of extended BBB disruption. Such experiments could also predict potential adverse effects of e.g. local FUS-induced inflammation or molecular effects from particular drug uptake and metabolism allowing for effective selection of most promising drug candidates.

While moving to larger target opening volumes and drug delivery, the heterogeneity of the BBB in the brain must also be considered [104, 105].

Recent single-cell RNA-seq studies revealed molecular heterogeneity of endothelial and mural cells found within different vascular segments and brain regions [105–108]. This transcriptomic heterogeneity of the NVU may have an implication for regional responses to FUS+MB as for example, BMECs found in hippocampus showed enrichment in inflammatory response and cell death genes as compared to cortex BMECs, indicating higher susceptibility of the hippocampus to vascular inflammation and injury [105]. Therefore, it is possible that observed segmentation of the cerebrovascular tree can affect vascular responses to FUS and MB parameters with certain brain regions being more susceptible to FUS+MB induced damage.

The NVU has also been shown to vary in the cellular architecture across different regions with a recent report demonstrating specialised BBB composition in the hippocampus enriched in the neural stem cells (NSC) [109]. In this study, Licht and colleagues identified the presence of NSC apical processes within NVU of the murine dentate gyrus that created direct connections with BMEC and led to selective uptake of the otherwise BBB-impermeable chemotherapeutic agent doxorubicin from the blood stream. With observed cognitive impairment as a side-effect of doxorubicin treatment, it is possible the same mechanism occurs in human brain [110]. This suggests that the cellular heterogeneity of the BBB in the brain can translate into region specific drug uptake and may have important implications for FUS-mediated drug delivery. With the hippocampus being strongly implicated in the pathology of dementia and therefore offering an attractive drug target region, development of new brain area-specific BBB *in vitro* models, e.g. hippocampal BBB enriched in neural stem cell components, may help elucidate regional responses of the BBB to FUS+MB and facilitate the most effective drug delivery strategy. Another example comes from PD where neurodegeneration is driven by progressive dysfunction of dopaminergic neurons in the substantia nigra – an area projecting to the cerebral cortex which was targeted by FUS+MB opening in a PD clinical trial [111] and in rat brain [112]. Recently, Pediaditakis *et al.* developed a novel substantia-nigra human Brain-Chip *in vitro* model containing dopaminergic neurons, astrocytes, microglia, pericytes, and BMEC that could serve as a testing platform for validation of new drugs for PD [113]. Such multicellular brain-region specific BBB models, when combined with FUS+MB *in vitro* system, could become useful predictors of FUS-mediated therapeutic uptake in disease-associated brain structures.

Finally, neurodegenerative disorders are one of the most heterogenous disease types with broad interpatient variability observed in disease onset, clinical symptoms, pace of progression, genetic mutations underlying the disease, and resulting responsiveness to treatment [114, 115]. This heterogeneity may be a key confound to understanding the patient’s BBB responses to FUS+MB and impede identification of one-size-fits-all FUS+MB parameters leading to safe BBB opening. With increasing speed of hiPSC reprogramming [116, 117] and expanding availability of *in vitro* BBB models derived from AD, PD, ALS and Huntington’s disease (HD) patient’s hiPSCs [76, 84], the high-throughput screening of sonication and MB effects on vasculature in specific patient or disease models may aid in personalization of FUS+MB treatment limiting the risk of side effects. This biological experimentation when combined with rapid technological progress in ultrasound devices will allow for tailoring of FUS sonication, MB type and drug treatment individually for the needs of specific patients to maximize the therapeutic effects of ultrasound in the clinic.

## Conclusions

FUS+MB mediated BBB opening, and consequent improved drug delivery, holds the promise to revolutionize neurodegenerative disease treatment.

Despite the profound preclinical and clinical progress in recent years, it still remains unknown whether localised BBB opening in human brain would indeed lead to increased drug uptake, how this would affect pharmacokinetics of drugs in the neurodegenerative brain environment, and if overall improved therapeutic outcomes will be achieved.

Novel patient-derived *in vitro* BBB models may aid in answering at least some of these questions, allowing for high-throughput FUS+MB parameters and drug treatment screening in the disease and genotype specific manner. Together, interdisciplinary efforts encompassing *in vitro* investigations, well designed clinical trials, and biomedical engineering will lead to safe and effective BBB opening in patients, paving the way towards successful translation of FUS+MB technology in neurodegenerative diseases.

## Data Availability

Not applicable.
